# Opioid Analgesics after Bariatric Surgery: A Scoping Review to Evaluate Physiological Risk Factors for Opioid-Related Harm

**DOI:** 10.3390/jcm12134296

**Published:** 2023-06-27

**Authors:** Stephanie C. M. Wuyts, Bart Torensma, Arnt F. A. Schellekens, Cornelis (Kees) Kramers

**Affiliations:** 1 Pharmacy Department, Universitair Ziekenhuis Brussel (UZ Brussel), 1090 Brussels, Belgium; 2 Research Group Clinical Pharmacology and Clinical Pharmacy, Faculty of Medicine and Pharmacy, Vrije Universiteit Brussel, 1090 Brussels, Belgium; 3 Department of Anesthesiology, Leiden University Medical Center (LUMC), 2333 ZA Leiden, The Netherlands; bart@torensmaresearch.nl; 4 Department of Psychiatry, Radboud University Medical Center, 6525 GA Nijmegen, The Netherlands; arnt.schellekens@radboudumc.nl; 5 Department of Internal Medicine and Pharmacology-Toxicology, Radboud University Nijmegen Medical Center, 6525 GA Nijmegen, The Netherlands; kees.kramers@radboudumc.nl

**Keywords:** morphine, pharmacology, opiate addiction, prescription drug abuse, substance use disorders

## Abstract

The persisting use of opioids following bariatric surgery has emerged as a prevalent complication, heightening the probability of opioid-related harm (ORM), such as opioid-related fatalities and prescription opioid use disorder (OUD). A comprehensive review of PubMed literature from 1990 to 2023 was conducted to pinpoint physiological influences on postoperative ORM. As a result, we found that patients undertaking bariatric operations often exhibit an inherently higher risk for substance use disorders, likely attributable to genetic predisposition and related neurobiological changes that engender obesity and addiction-like tendencies. Furthermore, chronic pain is a common post-bariatric surgery complaint, and the surgical type impacts opioid needs, with increased long-term opioid use after surgeries. Additionally, the subjective nature of pain perception in patients with obesity can distort pain reporting and the corresponding opioid prescription both before and after surgery. Furthermore, the postoperative alterations to the gastrointestinal structure can affect the microbiome and opioid absorption rates, resulting in fluctuating systemic exposure to orally ingested opioids. The prospect of ORM development post-bariatric surgery appears amplified due to a preexisting susceptibility to addictive habits, surgically induced pain, modified gut–brain interaction and pain management and the changed pharmacokinetics post-surgery. Further research is warranted to clarify these potential risk variables for ORM, specifically OUD, in the bariatric population.

## 1. Introduction

Trends of increased chronic prescription opioid use for non-cancer pain raise concerns not only in the United States and Canada but also in European countries [1,2]. Although these drugs are very effective analgesics, especially for cancer pain treatment [3], the evidence base for their use in (chronic) non-cancer pain is limited [4]. At present, geographical and practice variations in opioid prescribing are high [5,6], potentially indicating inappropriate use but complicating a thorough evaluation of the exact societal burden. Nevertheless, there is a confirmed risk for nonfatal overdose, morbidity and mortality [7,8]. In addition to other harms such as cardiovascular and endocrine complications, chronic opioid use is also associated with opioid use disorder (OUD) development [9,10].

In patients undergoing surgery, opioids are still the mainstay for peri- and postoperative pain management, despite their known side effects and risks [11]. Describing the public health impact of persistent postoperative opioid use remains difficult due to several study limitations. In the systematic review by Sitter and Forget, differences in reported opioid user rates could not be appointed to a specific surgery type, and unfortunately, the data on long-term follow-up were limited [12,13]. In a comparison of new persistent postoperative opioid use in patients after minor versus major surgeries, the rates were comparable (5.9% to 6.5%, adjusted odds ratio (OR), 1.04; 95% confidence interval (CI) 0.93–1.18) [14]. However, patients having major surgery, such as bariatric surgery, do seem to have a high risk for persistent postoperative opioid use (up to 9.9%) [14].

Bariatric surgery involves an invasive intervention targeting the gastrointestinal system. The conventional and most common interventional procedures are Roux-en-Y gastric bypass (RYGB) surgery and sleeve gastrectomy (SG) [15,16], representing, respectively, 29.3% and 55.4% of all bariatric surgery procedures according to the 2018 survey of the International Federation for the Surgery of Obesity and Metabolic Disorders [16]. After RYGB, the stomach is left in two parts: an upper pouch, anastomosed to the jejunum, and a lower gastric remnant. This biliopancreatic limb is anastomosed to the bowel further from the gastrojejunostomy to facilitate the passing of biliary fluids. An SG procedure involves a vertical transection of the stomach, leaving a tiny bag [15]. In earlier days, laparoscopic gastric banding (LAGB), a less invasive procedure, was also used to reduce the stomach’s volume using an adjustable band placed below the gastroesophageal junction [15,16]. The latter procedures are restrictive bariatric surgeries, whereas RYGB also involves the malabsorption of nutrients due to the diverted flow of bile and pancreatic enzymes [15].

Both new and prolonged opioid use are important complications of bariatric surgical procedures [17]. Patients with obesity using prescription opioids preoperatively are more likely to continue these drugs after bariatric surgery than opioid-naïve patients, which is not without risks [18,19,20,21,22]. The perioperative use of opioids in bariatric surgery results in worsened clinical outcomes (e.g., a more extended hospital stay and increased episodes of apnea) and in particular persistent opioid use post-discharge [17,23,24,25,26].

The prospective Longitudinal Assessment of Bariatric Surgery−2 (LABS-2) study by King et al. described a preliminary decrease in prescription opioid use from 14.7% to 12.9% in the initial postoperative phase (n = 2258). This was confirmed by Iranmanesh et al. in a retrospective study evaluating 11,179 Canadian patients prescribed opioids after bariatric surgery [27]. Nevertheless, over time, the prevalence of opioid use in patients who were opioid-naïve preoperatively in the LABS-2-study (n = 1892) increased from 5.8% at six months to 14.2% at seven years [20]. This persistent increase in opioid use has been directly attributed to the surgical intervention in a Swedish matched case–control study comparing opioid use following surgery to opioid use after an intensive lifestyle intervention (16.9% versus 9.0% at 8 years post-intervention) [28].

Approximately 10% of bariatric surgery candidates have a preoperative SUD history, which is positively correlated with postoperative SUD [29,30]. These patients have a 2.47 higher overall mortality risk postoperatively, due to either internal (Hazard Ratio (HR) = 2.29, *p* < 0.01) or external causes (HR = 3.16, *p* < 0.01), than patients without a previous SUD diagnosis [31]. Patients may have a significant risk reduction for new-onset SUD after bariatric surgery compared to patients with overweight or obesity [32]. A subset of patients develop new-onset SUD (e.g., tobacco, alcohol or illicit drugs), the proportions ranging from 6.55% to 89.5% [32,33,34]. These patients appear to have an increased susceptibility toward postoperative SUD, originating from underlying individual risk factors [34] notably seen in bariatric surgery.

The meta-analysis of Lin et al. identified that almost 90% of patients are prescribed excessive opioids following bariatric surgery [35], leaving them actively exposed to substances with a high potential for SUD development. Patients at risk for narcotic abuse did not show higher morphine equivalents prescribed at discharge compared to low-risk patients [36]. This indicates that not only the number of prescribed morphine equivalents or baseline abuse risk but other factors should be considered in order to reduce the harms and costs related to the development of opioid-related harm, particularly prescription OUD, in bariatric patients. Insight into the mechanisms contributing to this risk is crucial.

This scoping review aims to provide an overview of the potential physiological factors associated with altered opioid exposure and susceptibility after bariatric surgery to identify areas for future research to prevent opioid-related harm and OUD in this patient population.

## 2. Materials and Methods

A PubMed search was performed from 1 January 1990 to 17 May 2023. The search query consisted of the following MeSH terms: obesity, bariatric surgery, opioid analgesics, pharmacokinetics and pharmacodynamics, linked with Boolean operators. Grey literature was also searched, and a reference crosscheck was performed to detect eligible articles that were not identified through previous searches. The search was conducted without publication date or language restrictions.

All human studies on opioid use in adult patients with obesity and bariatric surgery were eligible if opioid exposure in the conditions pre- or post-bariatric surgery was evaluated. Despite the lower level of evidence, descriptive studies, cross-sectional studies, case series and case reports were included. Studies focusing on psychological and social factors were not included.

A two-stage screening process was followed to assess the eligibility for the inclusion of studies identified in the search. First, titles and abstracts were screened for relevance by one reviewer (SW) based on the inclusion and exclusion criteria. Next, full texts were screened by the same reviewer. Finally, all identified articles were checked by another reviewer (BT) and excluded if considered not relevant.

Data from full-text articles were extracted into categories of potential physiological factors increasing the risk for prescription OUD after bariatric surgery.

## 3. Results

Physiological factors were identified that may increase the patient’s susceptibility to develop long-term prescription opioid use and opioid-related harm. These can be categorized into premorbid factors, factors related to the surgical procedure and post-surgical care and altered pharmacokinetics.

### 3.1. Premorbid Factors

Several genetic risk factors for OUD development have been identified, as heritable factors significantly contribute to the risk to develop OUD (OUD heritability (h^2^) = ~0.50) [37]. Different gene panels, such as the sixteen single-nucleotide polymorphisms (primarily concerning opioid and dopamine receptors) in the Neuro Response prediction algorithm [38], have been studied to predict the baseline risk for OUD and therefore guide physicians in safe opioid prescribing.

After bariatric surgery, the dopamine equilibrium might be disturbed, as food cannot be used as a primary reinforcing stimulus, leaving a patient exposed to prescription opioids at risk of turning towards substances inducing a similar effect. Increases in dopamine concentrations in specific brain areas are caused by reinforcing cues such as drug consumption or energy-rich food intake [39,40]. The overlapping character of the ‘dopamine motivational system’, as referred to by Volkow et al., can either result in obesity by overeating or in drug addiction [40]. Blum et al. also discussed the hypothesis of an ‘addiction transfer’ [39]. The authors called this phenomenon the result of the ‘Reward Deficiency Syndrome’. As food addiction shows neurochemical similarities to SUD, overeating and, hence, obesity are the result of a blunted reward circuitry and weak satiety signal [39]. In their study, a large part of patients with obesity had disrupted dopaminergic neurotransmission caused by the presence of the Taq I DRD2 A1 allele (74% in obese subjects versus 24% without comorbid SUD), which is assumed to be a predictive genetic factor for the Reward Deficiency Syndrome [39].

### 3.2. Factors Associated with the Surgical Procedure

Chronic pain is described as the most frequent complaint of patients after bariatric surgery [20,41,42]. Though the etiology of, for example, abdominal pain has not been fully elucidated [41], the type and technique of the surgical intervention does affect the analgesic opioid requirement. Laparoscopic procedures have better outcomes than open surgery, because they result in a shorter length of stay and less opioid requirement [23]. In a retrospective matched case–control study, patients had a higher incidence of chronic opioid use in the 1–5 years following open RYGB (HR = 1.22, 95% CI: 1.06–1.41) than after laparoscopic RYGB (HR = 1.19; 95% CI: 1.06–1.34). Laparoscopic sleeve gastrectomy resulted in an even higher incidence of chronic opioid use (HR = 1.28; 95% CI: 1.05–1.56) [43].

Additionally, restrictive bariatric procedures modify the gut microbiota, its composition and circulating metabolites from intestinal bacteria [44]. Alterations in the gut microbiota can directly influence gut–brain communication and, consequently, the reward and stress response with a risk of OUD development [45,46]. It has already been shown that chronic morphine use can cause bacterial translocation across the gut wall and induce inflammation, which regulates brain function and, thus, may affect opioid addiction risk [46].

Secondly, the number and type of subsequent surgical procedures, especially cosmetic procedures, are considered a risk factor for continued opioid use [20]. For example, a thighplasty, which is a cosmetic surgical procedure that will tighten and improve the appearance of the thighs and is frequently requested by post-bariatric patients, resulted in a substantially higher risk for opioid use compared to a breast reduction (OR 3.66, 95% CI 1.24–10.84) in the study by Bennett et al. [47]. Their results also showed that 6.1% of opioid-naïve patients have persistent opioid use at least three months after cosmetic surgery. Those patients who were exposed to high-risk prescribing (e.g., long-acting opioid prescription, multiple prescribers, more than 100 oral morphine equivalents per day and overlapping treatment with benzodiazepines or other opioids) were at a higher risk for persistent use [47].

### 3.3. Pain Perception in Patients with Obesity

It has been suggested that obesity may be a risk factor for increased pain thresholds, which may result in a decreased need for pain control after surgery [48]. Currently, the literature is ambiguous about the effect of severe weight loss on pain perception, as discussed in a systematic review (2016) on pain experience and perception in patients with obesity before and after surgery-induced weight loss [48]. Other factors might be associated with a different pain perception, which was tested in a study in 2017 comparing pain sensitivity and pain scoring in patients with obesity to controls who did not have obesity [49]. The participants received multiple random thermal and electrical stimuli to the skin. Within the group of patients with obesity, pain stimuli were graded randomly, compared to patients without obesity who had more consistent scores [49], complicating pain assessment.

### 3.4. Pharmacokinetic Changes after Bariatric Surgery

Pharmacokinetics (PK) is how the body affects a specific chemical after administration through absorption, distribution, metabolism and elimination [23,41,50,51,52]. For orally administered drugs, the total bioavailability depends on the fraction that is absorbed from the gastrointestinal tract and escapes metabolism in the gut and liver. Anatomical changes to the gastrointestinal structure, caused by bariatric surgery, directly induce significant alterations in drug absorption [51,53], as shown in Table 1, while the subsequent weight loss, a more latent consequence, alters the liver metabolism [51], changing the maximal concentration (C_max_), time to maximal concentration (T_max_) or total exposure (Area Under the Curve (AUC)) [54,55,56]. As a consequence, the PK parameters also influence a potential drug addiction [57].

Until now, the literature on opioid PK after bariatric surgery has been limited. Table 2 presents an overview of the current PK studies of oral opioids in humans. As patients are prescribed different types of opioids after bariatric surgery, the generalizability of the described (side) effects is limited [50]. Most data concern morphine kinetics and indicate altered PK in the direct and latent post-surgery phases.

After administration of an oral morphine solution, a 2–7.5-times shortened T_max_ was measured in RYGB patients at 7–15 days and six months post-operatively, respectively. C_max_ increased up to 3.3-fold. Both the distribution and clearance decreased with the Body Mass Index (BMI), resulting in a significant increase in AUC_0-inf_ (+83%), with a 50% reduction of T_max_ (1 h to 0.5 h) [58]. Contrarily, Halcon et al. did not find significant changes in morphine C_max_ or AUC upon administration of a sustained release preparation in a matched controlled PK study of 12 women two years after surgery [59].

Increased exposure to the two significant metabolites of morphine, morphine-3-glucuronide (M3G) and morphine-6-glucuronide (M6G), was described by Lloret-Linares et al. in patients with obesity compared to patients at a healthy weight. They attributed this effect to a high morphine metabolic ratio and subsequently increased morphine glucuronidation in patients with obesity [64]. After RYGB, the (M3G + M6G)/morphine metabolic ratio decreased, as confirmed in a PK study at six months postoperative versus the preoperative period (−26%; range −74% to +21%; *p* = 0.004). Associated factors were the BMI, fat mass (kg) and triglyceride levels. On the other hand, in the immediate postoperative period, a temporary increase in this metabolic ratio was described [64].

PK alterations of opioids other than morphine after gastric surgery have not been studied extensively. Controlled-release oxycodone appeared to be absorbed in a shorter period (T_max_ 11.5 min versus 14 min) in RYGB patients compared to healthy volunteers at 1 year post-surgery. The AUC increased 14.4% [60]. Contrarily, a PK study on non-obese cancer patients also studied an oral oxycodone prolonged release preparation after total gastrectomy and did not find differences in the PK parameters [61]. Two case reports of patients on opioid replacement therapy with buprenorphine [62] and methadone [63] were identified, reporting increased T_max_ and C_max_ in the month following surgery. For buprenorphine, a decreased AUC (up to 43% at 1 year post-surgery) was determined [62]. In the methadone case, the AUC increased significantly (up to 213% at seven months post-surgery) [63].

## 4. Discussion

This is a scoping review of physiological factors contributing to the increased risk of opioid-related harm and OUD in patients undergoing bariatric surgery. The increased risk of chronic opioid use and OUD development might be due to premorbid risk factors for the development of addictive behavior, postoperative pain, altered pain perception and due to pharmacokinetic changes after surgery. However, the level of evidence is still low and requires more elaboration.

### 4.1. Risk Factor Assessment

Personalized analgesia should be provided based on a baseline risk assessment [65]. Testing the patient’s previous addictive behavior is essential before bariatric surgery, including extensive family history anamnesis, as more and more evidence is arising on the shared heritability of SUD, including OUD [38]. The role of neurotransmitters in specific brain areas is being explored to identify patients at risk for addiction transfer [39]. in addition to dopamine neurotransmission, the role of the mu-receptor has also been studied in patients during a bariatric procedure, because this receptor availability may be a marker for persistent weight loss [66]. In a study with rats, a downregulation of mu-opioid signaling was demonstrated following RYGB in stress- and energy-regulating brain regions, which may increase stress sensitivity and can potentially influence addictive behavior [67,68]. However, this was contradicted in studies with humans, because mu-opioid receptors were upregulated in bariatric patients after weight loss [69]. This illustrates the fact that, to date, neurotransmitters and their associated receptors and signaling are important but not well understood in opioid addiction research. Increasing the research on shared genetics and neurobiological processes influencing the human brain after bariatric surgery to define the potential effects on opioid PD in this specific patient group is necessary.

Since the gastrointestinal tract is extensively changed after bariatric surgery, postoperative pain is common, and subsequent surgical procedures are frequently required [41,43,47]. In a review and meta-analysis by Chang et al. (2014), an overall complication rate of 17% (95% CI: 11–23%) was established among US patients undergoing bariatric surgery. The authors showed that, although RYGB is the most effective procedure for weight loss compared to SG and LAGB, this type of surgery has the highest complication rate (21% versus 13% in SG and LAGB) [70]. Therefore, early pain detection following bariatric surgery is necessary, as this could increase the patient’s access to opioids for pain treatment but may also directly influence the addiction risk [23,24,43]. The anatomical changes also induce a neurobiological effect at the level of the microbiome [45]. After surgery, the gut peptides and bile acid metabolism are changed, which results in altered gut–brain axis communication. Even opioid drugs appear to influence the bacterial flora [46]. These effects are becoming essential to consider when evaluating (food) cravings, depression, satiety, self-esteem and, thus, their role in OUD [71,72,73,74,75,76]. More insights are needed on the effect of the surgical intervention, on the one hand, and chronic opioid exposure on the other, to determine the overall effect on opioid addiction following bariatric surgery.

Unfortunately, several variables are still insufficiently known, such as the pain’s etiology pathways [41], as well as how they affect opiate consumption and addiction of patients after a bariatric procedure. Data on health-related quality-of-life factors show an improvement in bodily pain and physical function after bariatric surgery [77,78], but the incidence of these factors remains high at long-term follow-ups (range 41–72% at 7 years post-intervention) [77]. A study by Björklund et al. described a counterintuitive effect of morphine on the muscular tone in the Roux limb of RYGB patients, potentially causing abdominal pain. They hypothesized that morphine can be a cause of pain as well as a treatment, leading the patient into a vicious cycle of opioid use [79]. An additional unknown factor is the pain perception of patients with obesity. As patients with obesity tend to have a higher pain threshold than patients without obesity, a pain assessment is complicated, and this affects the treatment of the patient [49]. So far, excess body weight has been identified as a risk factor for differences in pain perception in patients with obesity versus without obesity [48].

Since the evidence on the underlying mechanisms is still scarce, more fundamental pain research should be conducted so physicians can be guided in pain perception and scoring in patients with obesity undergoing weight loss after bariatric surgery.

### 4.2. Pharmacokinetic Modelling in Bariatric Patients

Studies on PK models for other opioids than orally administered morphine are lacking and remain necessary to understand the underlying physiological processes influencing opioid PK in patients undergoing bariatric surgery. Evidence from other patient groups, such as gastric oncology patients, cannot be extrapolated because of different characteristics. The direct effects on absorption can be analogous in bariatric surgery, but the latent impacts on distribution and metabolism are completely different [61].

Studies on the PK behavior patterns of opioids in patients with obesity before and after bariatric surgery are therefore necessary, especially since the impact of PK alterations on prescription OUD risk is not well understood. Future PK study designs should be diverse and have focus points on the immediate (<1 year) and late periods (>1 year) following surgery, since the impact of a substantial amount of weight loss on the PK parameters should not be underestimated [41]. First, initial changes in lean body mass influence the drug’s distribution volume. Second, a continuous low-grade inflammation status is reversed, leading to a reduced production of acute-phase proteins and increased Cytochrome P (CYP) 450 enzyme expression in the proximal intestinal gut wall and liver [51]. Third, a decreased prevalence of non-alcoholic steatohepatitis and non-alcoholic fatty liver disease after bariatric surgery has been proven to influence lipid peroxidation, CYP metabolism, and hepatic drug transporters [41,51,80]. Finally, a reduced liver size and alterations in cardiac output can lead to a reduced clearance and, consequently, differences in drug elimination [23]. In the meantime, physicians can opt to prescribe transdermal or sublingual opioids in order to bypass the gut and liver, especially during the immediate postoperative period, to avoid the first-pass effect and facilitate goal-directed pain management.

### 4.3. Prescribing Drugs after Bariatric Surgery

The anatomical changes after bariatric surgery might induce the faster onset of a drug’s effect (short T_max_), a higher effect intensity (high C_max_) and influence the overall exposure (AUC), affecting the risk for drug dependence and abuse. This renders prescribing drugs for opioid management challenging in a patient after bariatric procedures. Evidence-based recommendations are still unavailable, although studies on oral morphine PK have resulted in the recommendation to reduce the morphine dose in case a morphine prescription is inevitable after bariatric surgery to avoid increased exposure [58,64]. When limiting the availability and, thus, the prescription of opioids to control OUD, pharmacological interventions should also focus on other analgesic drugs than opioids (e.g., paracetamol, lidocaine patches and gabapentin) and non-pharmacological alternatives. As such, the updated 2021 Enhanced Recovery After Bariatric Surgery (ERABS) Society recommends an opioid-sparing multimodal postoperative pain protocol for this type of surgery [81], which has shown to be as equally effective as an opioid-based protocol [24,81,82,83,84,85,86,87,88]. Additionally, specialized prevention programs can help patients who are at risk for SUD development post-surgery [89].

### 4.4. Limitations

As this was not a systematic review, certain relevant studies might not have been included, and no risk of bias and publication bias assessment of the included studies was performed. Moreover, only physiological factors that could influence opioid addiction were evaluated. Psychological factors, such as traumatic life experiences or emotional regulation difficulties, and social aspects, such as social isolation or financial problems, could also affect the risk for the development of prescription OUD, because it is known that bariatric patients are at risk for SUD, especially when they have poor coping skills or if they experienced major life events post-surgery [34]. A combined approach is essential because, although all-cause mortality appears to be reduced after surgery (HR = 0.84; 95% CI: 0.79–0.90), suicide rates remain high among young adult patients who had bariatric surgery compared to matched controls (HR = 2.40, 95% CI: 1.57–3.68) [90].

This scoping review could not fully address what pharmacological changes after bariatric surgery directly influence opioid management. Future prospective studies are needed in patients with obesity to evaluate their baseline risk for addictive disorders in general and prescription OUD in particular to determine the (neuro)pharmacological changes after bariatric surgery, providing a sufficiently long follow-up period to identify those patients showing relapsed or new-onset addictive behaviors. In the study reports, the proportion of patients with chronic prescription opioid exposure developing an OUD should be mentioned as well.

## 5. Conclusions

Patients undergoing bariatric surgery are at risk for the chronic use of prescription opioids and subsequent development of opioid-related harm and, potentially, prescription OUD. This scoping review focused on the physiological factors contributing to this risk and identified genetic predisposition, postoperative pain, microbiome changes, altered pain processing and pharmacokinetic changes after bariatric surgery as potential physiological risk factors contributing to the increased risk of developing prescription OUD in bariatric patients. There is, however, a relative paucity of studies on physiological factors affecting the risk for the development of prescription OUD in the bariatric population. Comprehensive pharmacokinetic knowledge is essential to estimate the onset, intensity and total exposure to prescription opioids, as this might guide preventive measures to provide adequate analgesia in these patients.

## 6. Future Directions

A threefold strategy is suggested to guide future research to ultimately develop targeted screening programs for prescription OUD prevention and provide evidence-based opioid dosing recommendations. First, one should focus on identifying potential physiological risk factors for opioid-related harm in bariatric patients in prospective, multi-center, multi-domain epidemiological trials. Secondly, it is crucial to gain more insights into the dynamics of opioid use after bariatric surgery, starting with the assessment of the fundamental principles on genetics, pain etiology and the microbiome. Thirdly, changes in the PK of different oral opioids pre- versus post-bariatric surgery should be studied more thoroughly in real life or using advanced modeling techniques, as well as their potential association with opioid tolerance, dependence and addiction risk. In the study designs of these trials, uniform definitions and sufficiently long follow-ups are of the essence.

## Figures and Tables

**Table 1:** Summary of alterations in drug absorption processes influencing oral drug availability, sorted by type of surgical procedure [53]. (↑ = increase; ↓ = decrease; - = no effect).

	LAGB ^1^	SG ^1^	RYGB ^1^
Gastric pH	-	↑	↑
Gastric volume	-	↓	↓
Gastric emptying	-	↑	↓
Exposure to gastric digestive enzymes	-	↓	↓
Small intestinal bacterial overgrowth	-	↑	↑
Intestinal surface area for absorption	-	-	↓
Intestinal transit time	-	-	↓
Exposure to metabolic enzymes and drug transporters in the intestinal wall	-	-	↓
Exposure to bile acids	-	-	↓
Enterohepatic cycle	-	-	↓

^1^ Abbreviations: LAGB = laparoscopic gastric band; SG = sleeve gastrectomy; RYGB = Roux-en-Y Gastric Bypass.

**Table 2:** Overview of the current opioid pharmacokinetic studies in humans undergoing bariatric surgery. (↑ = increase; ↓ = decrease; ? = unknown).

	Morphine [58]	Morphine [59]	Oxycodone [60]	Oxycodone * [61]	Buprenorphine [62]	Methadone [63]
Galenic oral formulation	Oral solution	Prolonged release	Oral solution vs. controlled release (lipid-based vs. water-swellable)	Prolonged release	Sublingual tablet	Oral capsule
Procedure	RYGB	RYGB	RYGB	Total gastrectomy	SG	SG
Study population	30 patients (men/women)	12 women	21 patients (men/women)	24 patients (men/women)	1 women (case report)	1 women (case report)
Controls	Before-after surgery	Matched controls	Reference PK/PD model in healthy volunteers	Healthy subjects (reference publications)	/	/
Blood sample timing	3 visits: * V0: before * V1: +7–15 days * V2: +6 months	12 samples 2 years after surgery	12 samples at least 1 year after surgery	12 samples in 5–12 days after surgery	4 visits: * V0: before * V1: +1 week * V2: +1 month * V3: +1 year	4 visits: * V0: 8 days before * V1: +5 days * V2: + 1 month * V3: +7 months
T_max_	* V0 vs. V1: ↓ (−2×) * V0 vs. V2: ↓ (−7.5×)	= (control: 3.0 (1.5–5.0) h vs. patient: 2.6 (1.4–6.0)	Controlled release: absorption lag time ↓ (control: 14 min vs. patient: 11.5 min) No PK differences between CR formulations	↓ (control: 3 h vs. patient: 2.2 h)	* V0 vs. V1: ↓ (0.8 h → 0.5 h) * V0 vs. V2: ↓ (0.8 h → 0.5 h) * V0 vs. V3: ↑ (0.8 h → 1 h)	* V0 vs. V1: ↓ (2.5 h → 1.5 h) * V0 vs. V2: ↓ (2.5 h → 1.5 h) * V0 vs. V3: ↓ (2.5 h → 1 h)
C_max_	* V0 vs. V1: ↑ (1.7×) * V0 vs. V2: ↑ (3.3×)	= (control: 16 (4–29) nM vs. patient: 11 (7–67) nM; *p* = 0.72)	No data	= (control: 10.6 vs. patient: 12.97 ng/mL)	* V0 vs. V1: ↑ (11.2 → 20.7 nmol/L) * V0 vs. V2: ↓ (11.2 → 8.1 nmol/L) * V0 vs. V3: ↓ (11.2 → 10.0 nmol/L)	* V0 vs. V1: ↑ (945 → 1414 nmol/L) * V0 vs. V2: ↑ (945 → 2128 nmol/L) * V0 vs. V3: ↑ (945 → 2564 nmol/L)
AUC	V0 vs. V1: ↑ (+23.4%) V0 vs. V2: ↑ (+55.5%)	= (control: 80 (34–156) nmol.hr/L vs. patient: 66 (32–406) nmol.hr; *p* = 0.71)	↑ (+14.4%)	?	* V0 vs. V1: ↓ (−6.3%) * V0 vs. V2: ↓ (−43%) * V0 vs. V3: ↓ (−42%)	* V0 vs. V1: ↑ (+41%) * V0 vs. V2: ↑ (+143%) * V0 vs. V3: ↑ (+213%)
Advice	Divide dose	No dose reduction	Bioequivalence demonstrated for both CR formulations	No dose reduction	Perioperative monitoring	Perioperative monitoring

Abbreviations: CR = controlled release; PD = pharmacodynamic; PK = pharmacokinetic; SG = sleeve gastrectomy; RYGB = Roux-en-Y Gastric Bypass; V = visit. * Total gastrectomy with Roux-en-Y reconstruction was performed in an oncologic setting and not for weight loss.

## Data Availability

All data generated or analyzed during this study are included in this article. Further enquiries can be directed at the corresponding author.
